# A key role for the novel coronary artery disease gene *JCAD* in atherosclerosis via shear stress mechanotransduction

**DOI:** 10.1093/cvr/cvz263

**Published:** 2019-10-04

**Authors:** Gillian Douglas, Vedanta Mehta, Ayman Al Haj Zen, Ioannis Akoumianakis, Anuj Goel, Victoria S Rashbrook, Lucy Trelfa, Lucy Donovan, Edward Drydale, Surawee Chuaiphichai, Charalambos Antoniades, Hugh Watkins, Theodosios Kyriakou, Ellie Tzima, Keith M Channon

**Affiliations:** 1 Division of Cardiovascular Medicine, Radcliffe Department of Medicine, BHF Centre of Research Excellence, University of Oxford, John Radcliffe Hospital, Oxford OX3 9DU, UK; 2 Wellcome Centre for Human Genetics, University of Oxford, Roosevelt Drive, Oxford OX3 7BN, UK; 3 College of Health and Life Sciences, Hamad Bin Khalifa University, Education City, Qatar Foundation, Doha, Qatar

**Keywords:** JCAD, Atherosclerosis, Shear stress, Endothelial cells, Kiaa1462

## Abstract

**Aims:**

Genome-wide association studies (GWAS) have consistently identified an association between coronary artery disease (CAD) and a locus on chromosome 10 containing a single gene, *JCAD* (formerly *KIAA1462*). However, little is known about the mechanism by which *JCAD* could influence the development of atherosclerosis.

**Methods and results:**

Vascular function was quantified in subjects with CAD by flow-mediated dilatation (FMD) and vasorelaxation responses in isolated blood vessel segments. The *JCAD* risk allele identified by GWAS was associated with reduced FMD and reduced endothelial-dependent relaxations. To study the impact of loss of *Jcad* on atherosclerosis, *Jcad*^−/−^ mice were crossed to an *ApoE*^−/−^ background and fed a high-fat diet from 6 to16 weeks of age. Loss of *Jcad* did not affect blood pressure or heart rate. However, *Jcad*^−/−^*ApoE*^−/−^ mice developed significantly less atherosclerosis in the aortic root and the inner curvature of the aortic arch. *En face* analysis revealed a striking reduction in pro-inflammatory adhesion molecules at sites of disturbed flow on the endothelial cell layer of *Jcad*^−/−^ mice. Loss of *Jcad* lead to a reduced recovery perfusion in response to hind limb ischaemia, a model of altered *in vivo* flow. Knock down of *JCAD* using siRNA in primary human aortic endothelial cells significantly reduced the response to acute onset of flow, as evidenced by reduced phosphorylation of NF-КB, eNOS, and Akt.

**Conclusion:**

The novel CAD gene *JCAD* promotes atherosclerotic plaque formation via a role in the endothelial cell shear stress mechanotransduction pathway.

**Time for primary review: 10 days**

## 1. Introduction

Genome-wide association studies (GWAS) have achieved significant progress in identifying, and robustly replicating, common genetic variation that contributes to increased coronary artery disease (CAD) risk. The post-GWAS challenge is to identify the genes that confer the causative association with the CAD locus and discover the biological mechanisms linking these genes to CAD. In particular, whilst many GWAS variants implicate genes with known or expected roles in processes that are central to our current understanding of CAD pathogenesis (e.g. lipid metabolism or inflammation), other GWAS variants implicate genes without a known functional role in CAD pathogenesis, with the potential to reveal new biological mechanisms.

We have previously identified a locus on chromosome 10 which harbours a single gene, *JCAD* (Junctional protein associated with Coronary Artery Disease; previously *KIAA1462*).^1^ This association has been robustly confirmed in subsequent meta-analyses.^2^ Using the STARNET data set, we identified an expression quantitative trait locus between the protective allele of the lead SNP rs2487928 which was associated with decreased expression of *JCAD* in both atherosclerotic and atherosclerosis free arterial tissue. This indicates that decreased expression of *JCAD* associated with the rs2487928 allele is protective,^3^ leading to the hypothesis that loss of *JCAD* would result in reduced atherosclerosis burden.

The function of the 1359-amino-acid protein encoded by *JCAD* is largely unknown. JCAD is an intrinsically disordered protein with no recognizable functional domains and little homology to other protein families. JCAD has been identified as a novel component of endothelial cell junctions where it is found to co-localize with VE-Cadherin.^4^ A role for *JCAD* has been identified in pathological angiogenesis with decrease vascular formation in response to matrigel and decreased tumour growth.^5^ We have recently identified *JCAD* as a negative regulator of Hippo signalling in endothelial cells, suggesting that JCAD may in part contribute to endothelial dysfunction by down-regulation of Hippo signalling, leading to increased YAP activity.^3^ These observations support the results from previous proteomic studies which implicated JCAD as an interactor in the Hippo pathway,^6^^,^^7^ and as a negative regulator of LATS2 kinase activity.^8^ Although these recent studies have increased our knowledge of the possible molecular functions of *JCAD*, our understanding of how *JCAD* alters the development and progression of atherosclerosis remains limited.

In this study, we sought to establish the role of *JCAD* in human vascular function and in the development of atherosclerosis. We reasoned a potential role for JCAD in endothelial cell function could mediate the known associations between CAD pathogenesis and endothelial cell responses to shear stress and blood flow. In support of this hypothesis, we found associations between the *JCAD* risk allele and endothelial function in CAD patients. In ApoE^−/−^ mice, loss of *Jcad* was associated with reduced atherosclerosis specifically in areas of disturbed blood flow and with reduced endothelial cell expression of pro-atherogenic cell adhesion molecules. In response to hind limb ischaemia, loss of *Jcad* impaired the recovery of blood flow in the ischaemic limb. Finally, *JCAD* knock down in endothelial cells altered the signalling response to acute changes in shear stress. These observations identify *JCAD* as a new CAD gene with a role in endothelial cell mechanotransduction, and provide a novel genetic link between CAD susceptibility and the response to altered blood flow.

## 2. Methods

### 2.1 Clinical studies

Patients were participants in the Oxford Heart Vessels and Fat (ox-HVF) cohort (www.oxhvf.com) undergoing elective cardiac surgery [*n* = 443, including valve replacement/repair or coronary artery bypass grafting (CABG)] at the John Radcliffe Hospital, Oxford University Hospitals NHS Trust. Exclusion criteria included any active inflammatory, neoplastic, renal, or hepatic disease. CAD was defined on a per-vessel basis (i.e. for each of the left main stem, left anterior descending, circumflex, and right coronary artery) on the basis of invasive coronary angiography, using at least two orthogonal angiographic projections, and the results of ischaemia testing, where these were performed. Significant CAD was defined as either a greater than 50% angiographic stenosis, and/or evidence of flow limitation if pressure wire studies were undertaken to measure fractional flow reserve (FFR < 0.80), and/or evidence of inducible ischaemia in the territory on a myocardial perfusion scan. C-reactive protein (CRP), interleukin-6 (IL-6), and TNF-α were measured in serum. The protocols of the studies complied with the Declaration of Helsinki, and all patients provided informed written consent. The demographic characteristics are presented in Supplementary material online, *Table S1*.

Flow-mediated dilatation (FMD) and endothelium-independent vasodilatations (EIDs) of the brachial artery were measured the day before surgery using a linear array transducer and automated off-line analysis (Vascular Analyser, Medical Imaging Applications LLC). For FMD measurement, brachial artery diameter was recorded before, and 60 s after a 5-min forearm blood flow occlusion. EID was assessed 3 min after a sublingual spray of glyceryl trinitrate (400 µg). FMD and EID of the brachial artery were defined as the % change in vessel diameter from baseline.

Vasomotor studies were performed in saphenous vein segments obtained during CABG, as previously described.^9^ The same anaesthetics were used in all cases, and each sample was always obtained at the same stage of the operation, to limit the between-patients variability. In brief, vessel rings were equilibrated in oxygenated (95% O_2_/5% CO_2_) Krebs–Henseleit buffer at 37°C to achieve a resting tension of 3 g. Vessel rings were pre-contracted with phenylephrine (3 × 10^−6^M); then endothelium-dependent relaxations were quantified using bradykinin (BK, 10^−9^M to 10^−5.5^M). Relaxations to the endothelium-independent NO donor sodium nitroprusside (SNP, 10^−10^M to 10^−6^M), were evaluated in the presence of the NOS inhibitor NG-nitro-L-arginine methyl ester (L-NAME; 100 μM).

### 2.2 Animals

C57BL6/N embryonic stem cell clones (JM8A3) harbouring a constitutive deletion in the *Jcad* gene (Jcad^tm1 (KOMP) Mbp^) were obtained from the KOMP Consortium (CSD46781). In these cells, exon 3 of *Jcad* was replaced with a lacZ reporter/neomycin selection cassette, resulting in the removal of over 90% of the coding region. Targeted ES clones were microinjected into C57BL/6J embryos which were transferred into pseudopregnant females. The resulting chimeras were bred with C57BL/6J mice. Heterozygous offspring were subsequently crossed with C57BL/6J mice for >8 generation, before intercrossing to obtain the homozygous mice and heterozygous/wild-type control litter mates. The generation and phenotyping of the knock-out model was carried out in accordance with Animal (Scientific Procedures) Act 1986, with procedures reviewed by the clinical medicine animal care and ethical review body (AWERB), and conducted under project licenses PPL 30/2457 and PPL 30/3080. Animals were housed in individually ventilated cages (between 4 and 6 mice per cage of mixed genotypes) in specific pathogen free conditions. All animals were provided with standard chow (B&K Ltd, UK) and water *ad libitum* and, maintained on a 12 h light: 12 h dark cycle at controlled temperature (20–22°C) and humidity. For atherosclerosis studies, female mice were fed a high-fat diet (HFD; SDS 829108 Western RD diet) from 6 weeks of age, for 10 weeks. Water and food were available *ab libitum*. Heart rate and systolic blood pressure was measured (between 9 and 11 am) using an automated computerized tail-cuff system in 16-week-old female mice, as described previously (Visitech BP2000, Visitech Systems Inc., USA).^10^ All mice were culled by exsanguination under terminal anaesthetic (isoflurane >4% in 95%O_2_ 5%CO_2_); depth of anaesthesia was monitored by respiration rate and withdrawal reflexes. All animal procedures were approved and carried out in accordance with the University of Oxford ethical committee and the UK Home Office Animals (Scientific Procedures) Act 1986. All procedures conformed to the Directive 2010/63/EU of the European Parliament.

### 2.3 Tissue collection

Tissue for histological analysis was collected from mice perfused with phosphate buffer saline (PBS) followed by 4% paraformaldehyde, tissue for biochemical analysis was collected from mice perfused with PBS only and was snap frozen in liquid nitrogen and stored at −80°C until analysis. Primary endothelial cells were isolated from lungs by immunoselection with CD31 antibody (BD Biosciences, UK) coated magnetic beads as described previously.^10^ Vascular smooth muscle cells (VSMCs) were isolated from the aorta by digestion after removal of the endothelial cell layer and the adventitia as described previously.^11^

Total RNA was extracted using the Ambion Pure Link kit animal studies and QIAamp DNA blood Midi kit (Qiagen, UK) for human studies. Quantitative real-time RT-PCR was performed with an iCycler IQ real-time detection system (BioRad Laboratories) for animal studies and a QuantStudio7 (ABI) for human studies using primers and probes from the TaqMan Gene Expression Assay system (Life Technologies). Gene expression data were normalized to an appropriate house keeper using the delta CT method.

### 2.4 Hind limb ischaemia

Sixteen-week-old male *Jcad^+/+^*, *Jcad^+/^*^*−*^, or *Jcad*^−/−^ mice underwent left femoral artery ligation to induce hind limb ischaemia. In brief, animals were anaesthetized with 2% isoflurane supplemented with oxygen, given preoperative analgesia (buprenorphine) and maintained at 37°C. The femoral artery was exposed aseptically and isolated from the vein and nerve, then ligated with 7-0 ligatures proximal to the bifurcation of the popliteal artery and distal to the lateral caudal femoral artery. Blood flow recovery to the ischaemic foot was sequentially monitored by colour laser Doppler in pre-warmed (5 min at 37°C) anaesthetized mice up to 14 days after ischaemia. The ratios of occluded over non-occluded values were compared. Muscle recovery was assessed in 5 µm paraffin sections of the gastrocnemius, 14 days after surgery in H and E stained sections. Areas of regenerative tissue (defined as muscle cells with a central nuclei) or necrotic (defined as hypereosinophilic muscle with no or swollen nuclei and the presence of a cellular infiltrate) was quantified using Image Pro Premier and expressed as a percentage of the total cross-sectional area.

### 2.5 Analysis of atherosclerosis

*Jcad* ^−/−^ mice were backcrossed with ApoE^−/−^ mice to generate *Jcad*^−/−^ ApoE^−/−^ and *Jcad*^+/^^−^ ApoE^−/−^ mice and *Jcad*^+/+^ ApoE^−/−^ controls. Female mice were fed a high fat for 10 weeks from 6 weeks of age. Biochemical analyses of plasma lipids were performed on heparinized blood plasma using enzymatic assays. Lesion size was assessed in paraffin-embedded aortic root sections stained with Masson’s-Goldner (Merck). Average lesion size was calculated from 6 sections taken at 45 µm intervals starting from the section showing all three aortic valve cusps. Lesion area, Gal-3 (R&D systems AF1197 1/250), smooth muscle cell α-actin (Merck A5691, 1/250), necrotic core and collagen positive areas were quantified from digitized microscopic images acquired using polarized light using Image-Pro Plus. Aortic plaque area was quantified by *en face* analysis of oil red O stained aortas. Aortic arches were dissected clean of fat and stained with oil red O in proprane-1, 2-diol for 1 h plaque area was quantified from digitized microscopic images using Image-Pro Plus.

Three-dimensional atherosclerotic plaques in the aortic arch were visualized using microCT. Arches were dissected free of fat and connective tissue and incubated in 2% phosphotungstic acid in PBS for 2 weeks at 4°C. Arches were embedded in 1.5% agarose and scanned at 5 µm resolution (70 kV and 142 µA with no filter) using a SKYscanner 1172 scanner (Bruker, Coventry, UK). Raw files were reconstructed using NRecon (Bruker).

### 2.6 *En face* immunohistochemistry

For *en face* analysis the inner curvature of the aortic arch from female mice was fixed for 40 min in 4% PFA, washed in PBS and permeabilized with 0.1% Triton X-100 for 10 min, and blocked with 10% normal goat serum in casein. Samples were incubated with either anti-CD54 (Biolegend, 116101) or, VCAM-1 (BD 550547), or the appropriate IgG at 1:50 dilution overnight at 4°C and the appropriate secondary, washed and further stained for β-Cadherin (BD Biosource, 610153) followed by DAPI, mounted and imaged with an Olympus confocal microscope (FV3000).

### 2.7 *In vitro* shear stress studies

Human aortic endothelial cells (HAECs) from healthy non-smoking donors were cultured in EGM-2 medium (Lonza). *JCAD* was knocked down by siRNA transfection for 72 h using smartPOOL siRNA (Dharmacon) and lipofectamine RNAimax (Invitrogen). For shear stress experiments, endothelial cells were transfected and plated onto slides coated with 10 μg/mL fibronectin. Cells were cultured overnight in M199 medium containing 0.5% FBS. Slides were loaded onto a parallel plate flow chamber in 0.5% FBS in M199 media and 12 dynes/cm^2^ of shear stress was applied for indicated times. Cells were lysed in RIPA buffer containing protease inhibitors (Roche), 10 µg/mL sodium orthovanadate and 1 mM phenylmethylsulfonyl fluoride (PMSF). Western blotting was carried out on cell lysates using standard techniques with antibodies for *JCAD* (Atlas, HPA017956), GAPDH (Millpore, MAB374), phospho-Akt (Ser473; 4060, Cell Signalling technology), phospho-eNOS (ser1177; 9571 Cell signalling Technologies), total eNOS (610296, BD), phosphor-NF-КB p65 (Ser536; 3031 Cell Signalling Technologies), and total NF-КB p65 (6956; Cell Signalling Technologies).

### 2.8 Statistical analysis

Data are presented as mean ± SEM. Normality was tested using the Shapiro–Wilk test. Groups were compared using the Mann–Whitney *U* test for non-parametric data or an unpaired Student’s *t*-test for parametric data. When comparing multiple groups data were analysed by analysis of variance (ANOVA) with Newman–Keuls post-test for parametric data or Kruskal–Wallis test with Dunns post-test for non-parametric data. When more than two independent variables were present a two way ANOVA with Tukey’s multiple comparisons test was used. When within subject repeated measurements were present a repeated measures (RM) ANOVA was used. A value of *P* < 0.05 was considered statistically significant. All experiments and analysis was carried out by personnel blinded to genotype. The experimental unit was defined as a single animal, animals of both genotypes were caged together and in all experiments animals of both genotypes were derived from more than one cage. Age- and sex-matched mice were randomly assigned to experiments.

For clinical studies, continuous variables were tested for normal distribution using the Kolmogorov–Smirnov test. Non-normally distributed variables were log-transformed for analysis. Continuous variables were compared by using one-way ANOVA followed by Bonferroni *post hoc* test when individual comparisons were applied. To interrogate the interaction of JCAD genetic locus variability with the presence of CAD and the number or diseased coronary vessels, multivariate linear regression analyses were performed where the presence of CAD and the number of diseased coronaries were used as dependent variables and the JCAD genotype (AA, AG, or GG) was used as an independent variable along with traditional vascular risk factors, namely age in years, sex, hypertension, hyperlipidaemia, diabetes mellitus, and smoking. Standardized beta coefficients (Bstand) are presented for each covariate in all models.

Analysis was carried out by personnel blinded to genotype. Only full datasets were included in each individual analysis, *n* numbers are detailed in figure legends. For the clinical study, we estimated that, given the expected frequency of the rs2487928 genotypes (∼20% AA vs. ∼35% GG in our cohort), a total of at least 265 patients would allow us to detect a difference in FMD of 1.85 with power 0.9 and SD = 3.3 between AA vs. GG. Regarding the association of rs2487928 genotypes with CAD, we estimated (given the frequency of the individual rs2487928 genotypes in our cohort) that with at least 430 patients we would be able to detect a difference of 0.21 in the hazard ratio for the presence of CAD between AA vs. GG with power 0.9.

## 3. Results

### 3.1 *JCAD* variants are associated with altered endothelial function in CAD patients

To test for associations between *JCAD* genotype and changes in vascular function, we genotyped 443 prospectively-recruited patients undergoing elective cardiac surgery for the *JCAD* eQTL SNP rs2487928. The *JCAD* risk allele for CAD was associated with the presence of CAD *β* = 0.127 (standard error = 0.022), *P* = 0.004, and with the number of diseased vessels as an indicator or disease burden, *β* = 0.136 (standard error = 0.066), *P* = 0.002. Both associations were independent of the traditional risk factors e.g. hypertension, hyperlipidaemia, age, and diabetes. These observations support the association between the *JCAD* rs2487928 risk allele and the presence of CAD.

**Figure 1:**
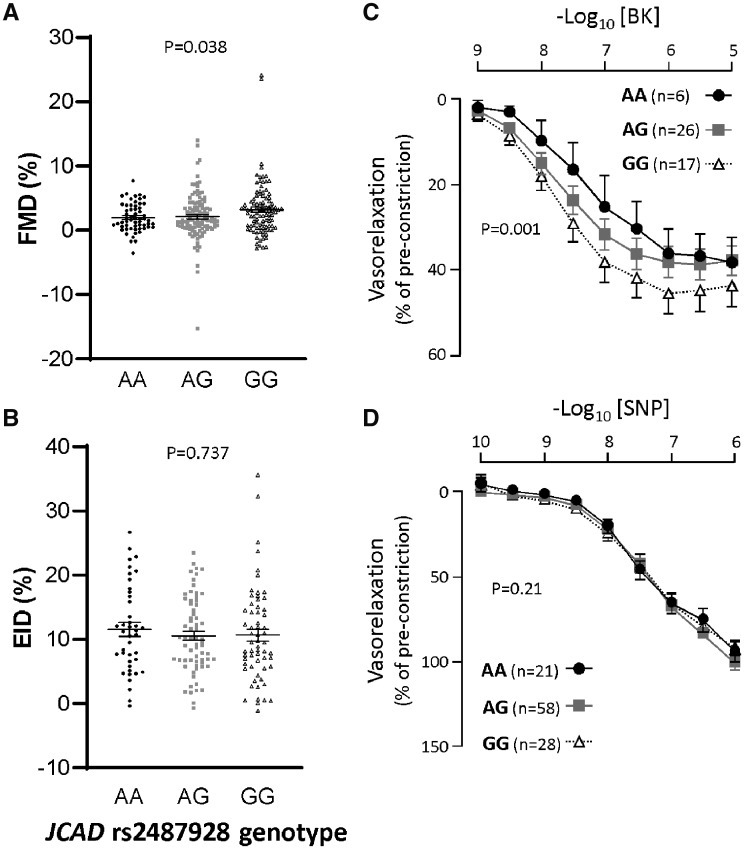
Carriers of the *JCAD* risk allele (AA) had significantly reduced endothelial cell function compared with carriers of the protective (GG) allele. (*A*) *In vivo* dilator response to flow (flow mediated dilation; FMD) was significantly greater in carriers of the protective (GG) allele compared with patients with the risk allele (AA; *P* < 0.05, one-way ANOVA; number of subjects AA = 58, AG = 115, GG = 100). (*B*) No difference between genotypes was observed in endothelial cell independent dilation (EID) in response to GTN *in vivo* (number of subjects AA = 41, AG = 66, GG = 61). (*C*) Saphenous veins from carriers of the protective allele had a greater sensitivity to bradykinin (BK) compared with carriers of the risk allele (*P* < 0.05, two-way ANOVA for repeated measures, number of subjects AA = 6, AG = 26, GG = 17). (*D*) Endothelial cell independent dilation in saphenous veins to sodium nitroprusside (SNP, number of subjects AA = 21, AG = 58, GG = 28) was not different between genotypes.

In order to test the influence of *JCAD* variants on endothelial cell function *in vivo*, we quantified brachial artery flow-mediated vasodilation using ultrasound measurement of brachial artery diameter before and after a brief occlusion of the vessel by suprasystolic inflation of a blood pressure cuff. Carriers of the *JCAD* rs2487928 risk allele had significantly reduced flow-mediated dilation responses compared with carriers of the protective allele (*Figure 1A*). This difference was not due to a change in sensitively of the VSMCs to nitric oxide, since endothelial cell independent dilation in response to GTN was not different between genotypes (*Figure 1B*). These *in vivo* studies were supported by *ex vivo* organ bath measurements of endothelial cell function in saphenous vein rings harvested at the time of cardiac surgery, revealing a significant decrease in sensitivity to the endothelial cell dependent vasodilator bradykinin in carriers of the *JCAD* rs2487928 risk allele (*Figure 1C*), with no difference in the response to the endothelium-independent dilator, sodium nitroprusside (SNP; *Figure 1D*). There were no differences in the plasma levels of either IL-6, TNF-α, or CRP between patients with different *JCAD* genotypes, indicating that differences in endothelial function were not related to possible effects of *JCAD* genotype on systemic inflammation (Supplementary material online, *Figure S1*).

### 3.2 Loss of *Jcad* does not alter baseline phenotypes or haemodynamic function

**Figure 2:**
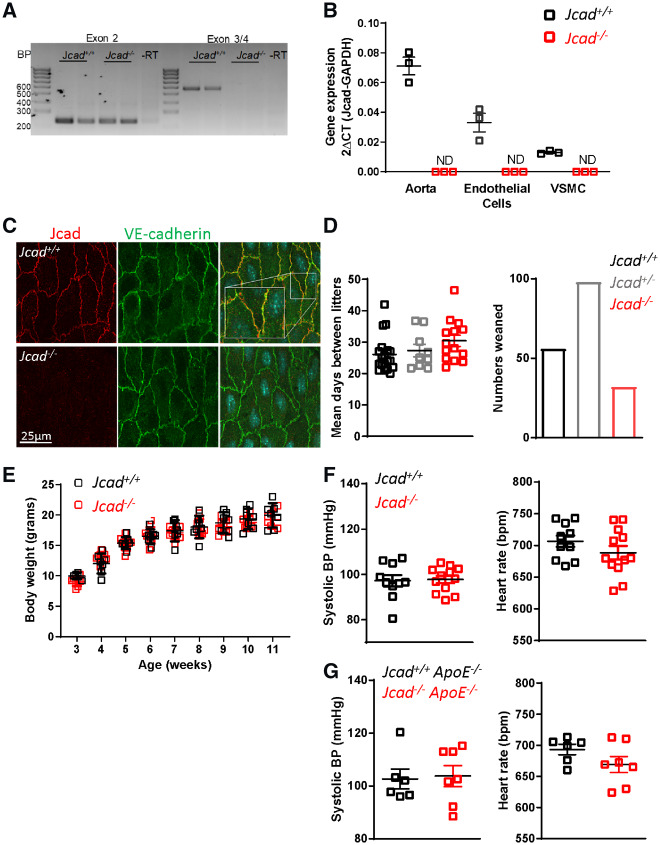
Characterization of the global *Jcad* knock out mouse. (*A*) PCR of aortic cDNA encoding exon 2 and exon 3–4 showing preservation of exon 3 and 4 in wild type (*Jcad^+/+^*) but not *Jcad* knock out mice (*Jcad*^−/−^). (*B*) Real-time qRT-PCR showing abundant expression of *Jcad* in aorta and primary endothelial and vascular smooth muscle cells (VSMC) from *Jcad^+/+^* mice but not detectable (ND) in samples from *Jcad*^−/−^ mice (*n* = 3 per group). (*C*) Representative images of *en face* staining for *Jcad* (red) and VE-cadherin (green) in descending aorta from *Jcad^+/+^* and *Jcad*^−/−^ mice, scale bar = 25µm. Zoomed in section to highlight junctional staining is shown in the white box. (*D*) No significant difference in days between litters with *Jcad^+/+^*, *Jcad^+/^*^−^ or *Jcad*^−/−^ breeding pairs (*n* = 9–19 litters per breeding pair). *Jcad*^−/−^ were born at the expected Mendelian ratio. (*E*) No difference was observed in body weight between *Jcad^+/+^* and *Jcad*^−/−^ female mice (*P* > 0.05, RM ANOVA) *n* = 5–8 per group. No significant difference was observed in systolic blood pressure or heart rate between female *Jcad^+/+^* and *Jcad*^−/−^ mice on either a (*F*) wild type (*ApoE^+/+^*, un-paired *T*-test, *n* = 9–13 per group)) or (*G*) hyperlipidaemic (*ApoE*^−/−^, Mann–Whitney, *n* = 6–7 per group) background (*P* > 0.05 unpaired *t*-test). Data are expressed as the mean ± SEM, each point represents an individual animal. Black symbols and bars = *Jcad^+/+^*, grey symbols and bars = *Jcad^+/−^*, red symbols and bars = *Jcad*^−/−^.

In order to investigate the mechanistic role of *Jcad* on CAD pathogenesis, we obtained global *Jcad* knock out (*Jcad*^−/−^) mice. cDNA from exon 2 was detected at the expected size in both WT and KO mice. However, as expected, bands were only observed in WT mice when primers spanning exons 3-4 were used, confirming loss of exon 3 in *Jcad*^−/−^ mice (*Figure 2A*). Real-time qRT-PCR confirmed vascular *Jcad* expression in the aorta and primary endothelial and VSM cells from *Jcad^+/+^* mice, with no expression observed in cells or tissues from *Jcad*^−/−^ mice (*Figure 2B*). *En face* immunofluorescent analysis of the endothelial cell layer in the descending aorta showed Jcad protein at endothelial cell junctions in close association VE-cadherin in *Jcad^+/+^* mice, that was completely absent in aortic *en face* preparations from *Jcad*^−/−^ littermates. The absence of *Jcad* did not appear to impact of VE-cadherin localization or expression, as staining appeared similar between genotypes (*Figure 2C*). *Jcad*^−/−^ mice were born at the expected Mendelian ratio with no difference in breeding efficiency observed between wild type and knock out breeding pairs. In addition, there was no difference in growth curves of female mice up to 11 weeks of age (*Figure 2E*), nor any difference in body weight or organ weights between genotypes in female mice at 16 weeks of age (data not shown). Loss of *Jcad* did not affect haemodynamic control as there was no difference in either systolic blood pressure or heart rate between wild type and *Jcad*^−/−^ mice (*Figure 2F*).

### 3.3 Loss of *Jcad* decreases atherosclerotic plaque formation

In order to assess the role of *Jcad* in atherosclerosis, we crossed *Jcad*^−/−^ mice with hyperlipidaemic ApoE^−/−^ mice. Breeding *Jcad^+/^*^−^ mice on an ApoE^−/−^ background revealed that *Jcad*^−/−^ offspring were significantly under-represented, with *Jcad*^−/−^ pups representing only 17% of the litter vs. 30% and 53% of the litter for heterozygous *Jcad^+/^*^−^ and *Jcad^+/+^* littermates (*P* < 0.05). However, in live born offspring there were no observable differences between *Jcad^+/+^* and *Jcad*^−/−^ ApoE^−/−^ mice in either body or organ weight at 16 weeks of age (Supplementary material online, *Table S2*).

To assess the role of *Jcad* in atherosclerotic plaque formation, we fed *Jcad*^−/−^ ApoE^−/−^ mice a HFD for 10 weeks from 6 to 16 weeks of age. Loss of *Jcad* did not affect plasma lipids, with no difference between genotypes observed in total, HDL or LDL cholesterol or triglyceride levels (Supplementary material online, *Table S3*). Furthermore, there was no difference in blood pressure or heart rate between genotypes after 10 weeks of high-fat feeding (*Figure 2G*).

**Figure 3:**
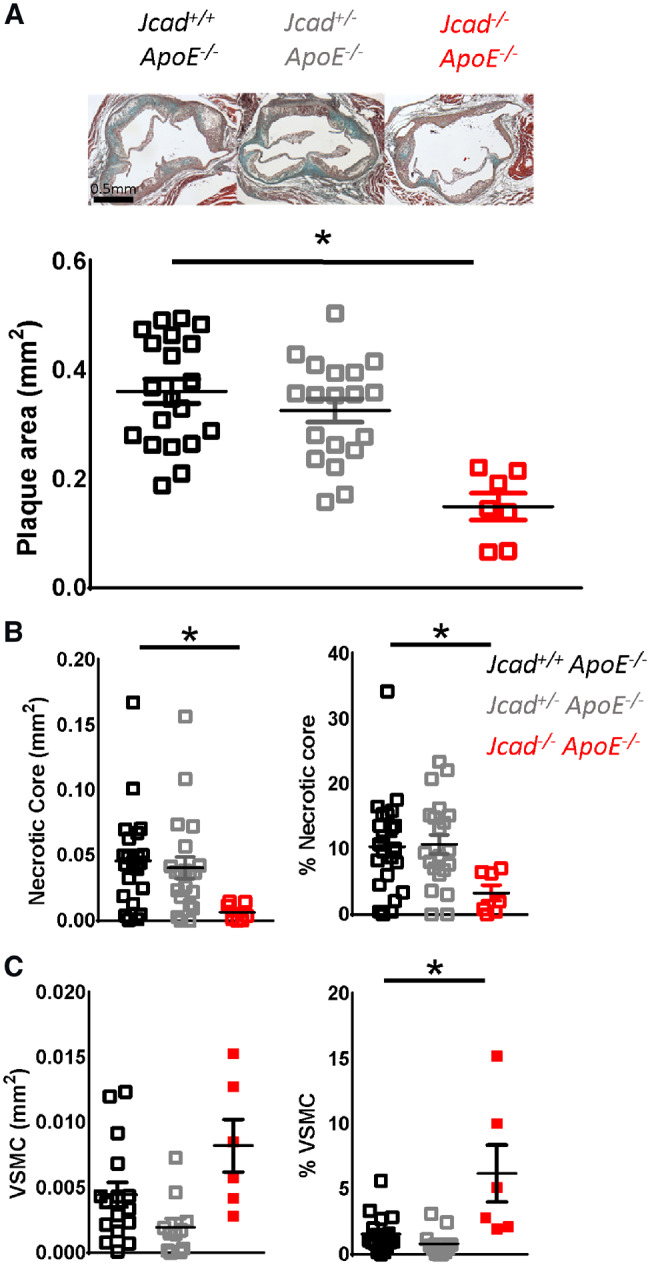
Loss of *Jcad* causes a significant reduction in atherosclerosis. (*A*) Representative images of aortic roots stained with Masson’s Goldner for plaque quantification in aortic roots of female *Jcad^+/+^ApoE*^−/−^, *Jcad^+/−^ApoE*^−/−^ and *Jcad*^−/−^*ApoE*^−/−^ mice fed a high-fat diet for 10 weeks, scale bar = 0.5 mm. A significant reduction in plaque area was observed in *Jcad*^−/−^*ApoE*^−/−^ mice (*P < 0.05, one way ANOVA, *n* = 20–7 per group). (*B*) A significant reduction was observed in both the absolute size of the necrotic core and when expressed as a percentage of plaque area in *Jcad*^−/−^*ApoE*^−/−^ mice (*P < 0.05, Kruskal–Wallis test, *n* = 20–7 per group). (*C*) A significant increase in the percentage of smooth muscle cell α-actin positive cells in plaques was observed in plaques from *Jcad*^−/−^*ApoE*^−/−^ mice (*P < 0.05, Kruskal–Wallis test, *n* = 17–6 per group). Data are expressed as the mean ± SEM, with each data point representing an individual mouse. Black symbols = *Jcad^+/+^* ApoE^−/−^, grey symbols = *Jcad^+/^*^−^*ApoE*^−/−^, red symbols = *Jcad*^−/−^*ApoE*^−/−^.

We first quantified atherosclerotic plaque in the aortic root of WT (*Jcad^+/+^*), heterozygous (*Jcad^+/^*^−^), and homozygous (*Jcad*^−/−^) mice after 10 weeks of HFD. We observed that *Jcad*^−/−^ mice had a significant decrease in atherosclerotic plaque in the aortic root (0.362 ± 0.02 vs. 0.15 ± 0.02 mm^2^ for *Jcad^+/+^ApoE*^−/−^ and *Jcad*^−/−^*ApoE*^−/−^, respectively; *Figure 3A*). Furthermore, loss of *Jcad* significantly decreased the area of the necrotic cores and increased smooth muscle cell content within atherosclerotic plaques (*Figure 3B* and *C*), consistent with a more stable plaque phenotype. Loss of *Jcad* was not associated with a difference in either macrophage or collagen content (Supplementary material online, *Figure S2*).

**Figure 4:**
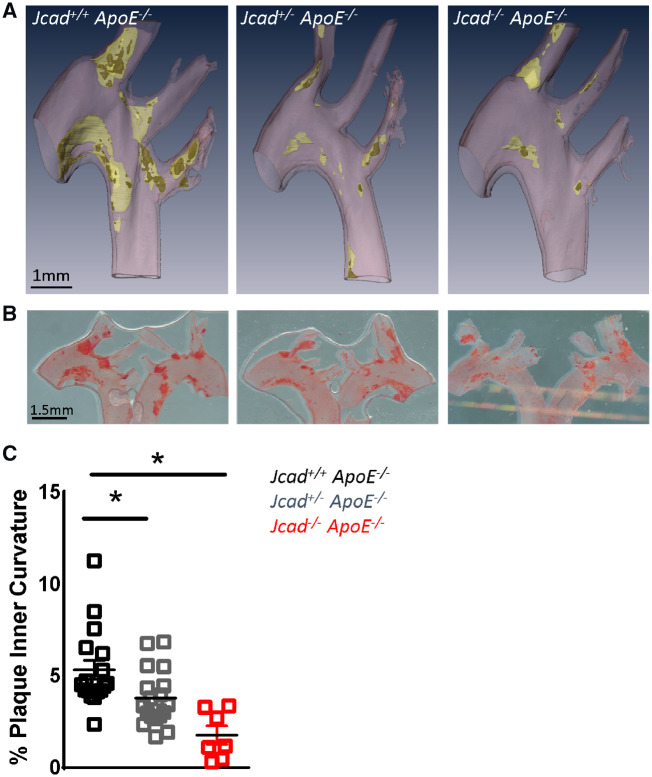
Loss of *Jcad* causes a significant reduction in atherosclerosis in the aortic arch inner curvature. (*A*) Representative three-dimensional renders generated from microCT images of atherosclerosis plaques in the aortic arch of hyperlipidaemic female wild-type (*Jcad^+/+^ ApoE*^−/−^), heterozygous (*Jcad^+/−^ ApoE*^−/−^), and homozygous (*Jcad*^−/−^*ApoE*^−/−^) knock out mice fed a high-fat diet for 10 weeks. Vessel wall is coloured pink, plaque yellow, and necrotic core dark yellow, scale bar = 1mm. (*B*) Representative images of the aortic arch stained for atherosclerotic plaques using Oil Red O lipid staining (plaque stains red; scale bar = 1.5 mm). (*C*) Quantification of *en face* plaque area in the inner curvature of the aortic arch reveals a significant reduction in plaque area in the inner curvature of *Jcad^+/^*^−^*ApoE*^−/−^ and *Jcad*^−/−^*ApoE*^−/−^ versus *Jcad^+/+^ ApoE*^−/−^ mice (*P < 0.05, Kruskal–Wallis test, *n* = 18–7 per group). Data are expressed as the mean ± SEM, each point represents an individual animal.

We next assessed how loss of *Jcad* altered atherosclerotic plaque formation in the aortic arch, as the formation and distribution of plaque in the aortic arch is dependent upon low or disturbed blood flow. We used microCT to assess plaque localization in the aortic arch. We observed that loss of *Jcad* resulted in a striking reduction in plaque burden in the inner curvature of the aortic arch (*Figure 4A*). To confirm this finding we quantified percentage plaque coverage in the inner curvature of the aortic arch using oil red O staining. Consistent with our microCT observations we found a significant impact of loss of *Jcad* on the development of atherosclerosis in the inner curvature, with progressive reduction in atherosclerosis observed between wild type (5.33 ± 0.52 mm^2^; *Jcad^+/+^ApoE*^−/−^*)*, heterozygous (3.80 ± 0.36 mm^2^; *Jcad^+/^*^*−*^*ApoE*^−/−^), and homozygous (1.78 ± 0.50mm^2^; *Jcad*^−/−^*ApoE*^−/−^) mice (*Figure 4B* and *C*).

### 3.4 Loss of *Jcad* decreases expression of inflammatory genes and proteins in areas of disturbed flow

**Figure 5:**
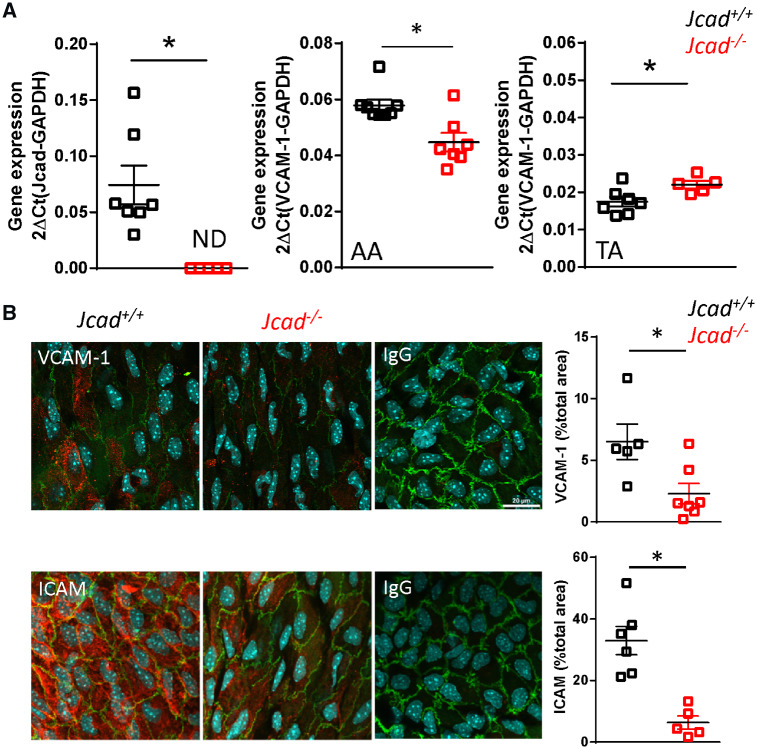
Loss of *Jcad* reduces expression of pro-inflammatory adhesion molecules in areas of disturbed flow. (*A*) Real-time qRT-PCR showing non-detectable (ND) expression of *Jcad* in aortas of female knock out (*Jcad*^−/−^) vs. wild-type (*Jcad^+/+^*) mice, *n* = 5–7 per group. Loss of *Jcad* causes a significant reduction in expression of *Vcam-1* in the aortic arch and a significant increase in expression in the descending aorta of *Jcad*^−/−^ mice (*P < 0.05, unpaired *t*-test, *n* = 5–7 per group). (*B*) Representative *en face* images and quantification of immunohistochemical analysis of Vcam-1 and Icam in the inner curvature of the aortic arch from *Jcad^+/+^* and *Jcad*^−/−^ mice, control sections were stained with the appropriate IgG control, scale bar = 20 µm. Loss of *Jcad* causes a significant reduction in the expression of Vcam-1 and Icam in the inner curvature (*P < 0.05 unpaired *t*-test, *n* = 5–7 per group). Data are expressed as the mean ± SEM, each point represent an individual animal. Black symbols = *Jcad^+/+^*, grey symbols = *Jcad^+/−^*, red symbols = *Jcad*^−/−^.

We next sought to assess the mechanisms linking loss of *Jcad* to protection from the development of atherosclerotic plaque in areas of low or disturbed flow. We first investigated whether loss of *Jcad* altered RNA expression of the pro-inflammatory adhesion molecule Vcam-1. We extracted mRNA from the aortic arch and thoracic aorta of *Jcad^+/+^* and *Jcad*^−/−^*ApoE^+/+^*mice. As expected expression of *Jcad* was not detectable in any samples from *Jcad*^−/−^*ApoE^+/+^* mice. However, expression of *Vcam-1* was significantly reduced in aortic arch samples from *Jcad*^−/−^*Apo*E^+/+^ mice on a wild type *ApoE^+/+^* background. In contrast a small but significant increase in *Vcam-1* expression was observed in the thoracic aortas from *Jcad*^−/−^*ApoE^+/+^* mice (*Figure 5A*). In order to establish the relationship of endothelial cells to the observed changes in Vcam-1, we used *en face* staining of the endothelial cell monolayer on the inner curvature of the aortic arch. A significant decrease in the endothelial cell immunofluorescence for both Icam and Vcam-1 was observed in the inner curvature from *Jcad*^−/−^*ApoE^+/+^* mice compared with their WT littermates (*Figure 5B*). The finding of decreased expression of pro-inflammatory adhesion molecules in areas of low/disturbed flow independent of plaque burden suggested that *Jcad* may play a role in the shear stress mechanotransduction pathway.

### 3.5 Loss of *JCAD* alters the cellular response to shear stress

**Figure 6:**
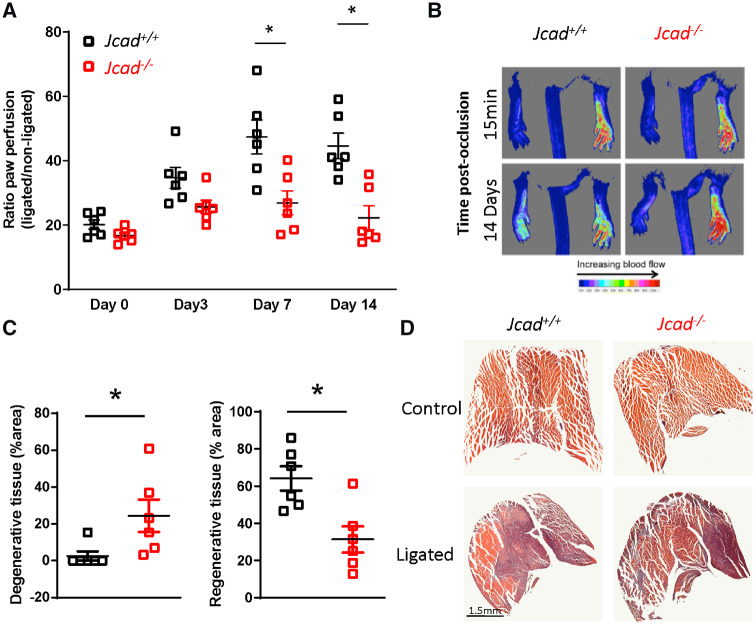
*Jcad* knock-out mice have a reduced recovery after hind limb ischaemia. (*A*) Reduced recovery of plantar perfusion in male *Jcad* knock out (*Jcad*^−/−^) mice after femoral artery ligation compared with wild types (*Jcad^+/+^*; *P < 0.05, RM ANOVA). (*B*) Representative Doppler images of plantar perfusion immediately after and 14 days post-femoral artery ligation (pseudocolor scale, arbitrary units). (*C*) Loss of *Jcad* is associated with increased presence of degenerative tissue (defined as hypereosinophilic muscle with no or swollen nuclei and the presence of multiple cellular infiltrate) and a decrease in regenerative tissue (defined as the presence of centralised nuclei) in gastrocnemius muscle 14 days after femoral artery ligation (*P < 0.05, Mann–Whitney). (*D*) Representative images of injured and un-injured gastrocnemius muscle 14 days after femoral artery ligation, scale bar = 1.5 mm. Data are expressed as the mean ± SEM, each point represent an individual animal, *n* = 6 per group. Black symbols = *Jcad^+/+^*, red symbols = *Jcad*^−/−^.

We next used a second model of altered blood flow to investigate the role of *Jcad* in the shear stress mechanotransduction pathway. Hind limb ischaemia by ligation of the femoral artery triggers shear stress-mediated adaptive remodelling of pre-existing thigh collaterals followed by angiogenesis in the downstream ischaemic muscle. As expected, ligation of the femoral artery caused an immediate reduction in plantar perfusion, as measured by Laser Doppler imaging, to a similar degree in both genotypes. In *Jcad*^−/−^ mice, the recovery of plantar perfusion was impaired compared to wild type mice, evident as early as Day 3 after femoral artery ligation, which persisted and reached significance at Day 7 and Day 14 (*Figure 6A* and *B*). As expected, this reduced perfusion resulted in impaired recover downstream of the ligation in the gastrocnemius muscle, with significantly greater necrotic muscle and reduce regenerative muscle observed 14 days post-ligation in *Jcad*^−/−^ mice (*Figure 6C* and *D*). A significant reduction in planter perfusion was also observed in heterozygous *Jcad*^*−*^^*/+*^ mice compared with their wild type littermates (Supplementary material online, *Figure S3*).

**Figure 7:**
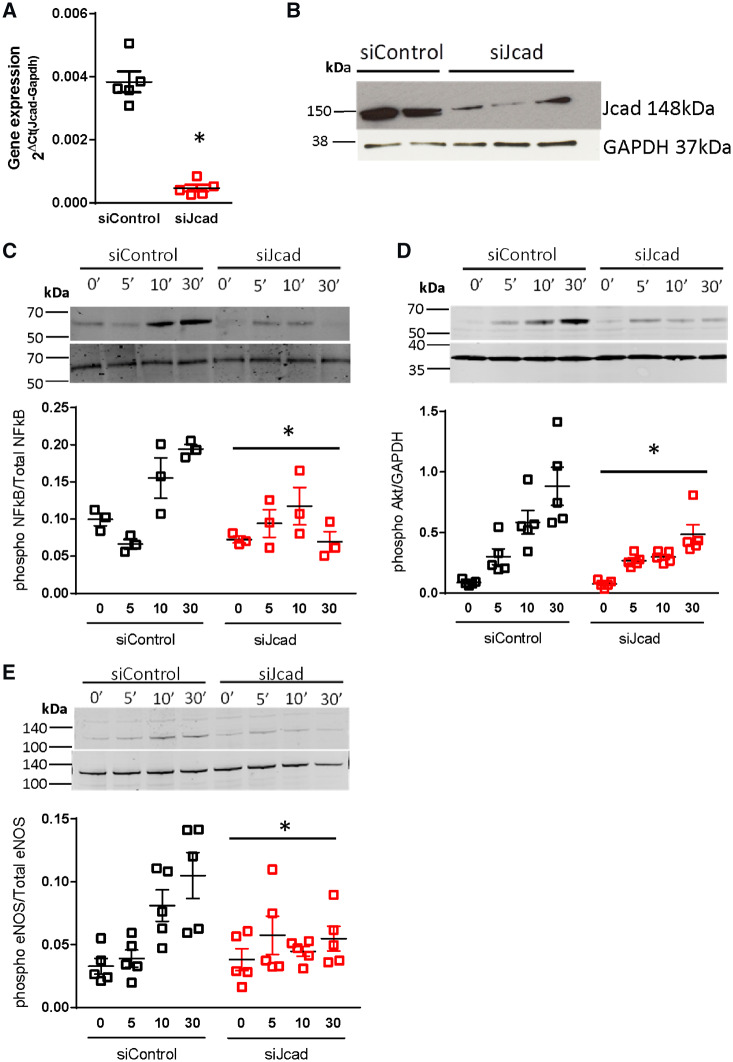
Knock down of *Jcad* in primary human aortic endothelial cells leads to an altered response to the onset of flow. siRNA mediated knock down of JCAD (siJcad) lead to a significant decrease in *JCAD* (*A*) mRNA (P < 0.05, unpaired *T*-test) and (*B*) protein expression (as determined by Western blotting) compared with control cells treated with non-targeted control siRNA (siControl). Control or JCAD knock down cells were plated onto fibronectin coated slides and exposed to shear stress (12 dynes/cm^2^) for the indicated times or kept as static controls (0–30 min). In siControl, a significant increase in phosphorylation was observed across the time course for all proteins studied (*P* < 0.05, RM ANOVA). Knock down of JCAD caused a significant blunting in the phosphorylation of (*C*) NF-КB p65, (*D*) Akt, and (*E*) eNOS in response to flow as assessed by Western blot, *P < 0.05, RM ANOVA across time points comparing siControl against siJcad, *n* = 3–5 per group.

Given that recovery of plantar perfusion is critically dependent on flow induced remodelling in collateral vessels,^19^ we reasoned that *Jcad* may play a role in the rapid cellular signalling responses to altered shear stress. To test this hypothesis, we studied the effect of *JCAD* knockdown in endothelial cells exposed to acute onset shear stress. Knockdown of *JCAD* with pooled siRNA achieved >80% reduction in *JCAD* mRNA and JCAD protein in HAECs (*Figure 7A* and *B*), compared with cell treated with non-targeted control siRNA. The onset of laminar flow (12 dyn/cm^2^) was associated with robust NF-KB phosphorylation (Ser536) in control cells. In contrast, no increase in NF-KB phosphorylation was observed in *JCAD* siRNA treated cells (*Figure 7C*). Furthermore, the decrease in Akt phosphorylation (Ser473) in response to the onset of flow in *JCAD* knockdown cells abolished the flow-dependent increase in eNOS phosphorylation (Ser1177; *Figure 7D* and *E*). Taken together, these observations demonstrate a requirement for *JCAD* in the acute response to shear stress in endothelial cells.

## 4. Discussion

*JCAD* was identified by GWAS studies as associated with CAD and myocardial infarction.^1^ We have now shown that loss of *JCAD* has a protective role in vascular function and atherosclerosis progression but is detrimental to recovery after hind limb ischaemia. Using an *in vitro* model of shear stress we demonstrate that the altered pathologies observed in these two *in vivo* models of altered flow are likely due to modulation of the shear stress mechanotransduction pathway by *JCAD*. Thus, we identify *JCAD* as a novel gene identified by CAD GWAS with a specific role in vascular cell mechanotransduction.

No study to date has investigated how *JCAD* genotype affects vascular function in humans. We confirmed that the *JCAD* risk allele is associated with increased CAD burden, as measured by the number of diseased vessels. We also show for the first time that the *JCAD* risk allele is associated with impaired endothelial cell function using both *in vivo* and *ex vivo* measurements. We found that in patients undergoing cardiac surgery, carriers of the *JCAD* risk allele attenuated vascular endothelial cell dependent vasomotor responses, as evidenced by both brachial artery FMD *in vivo*, and by the dilator response to bradykinin in *ex vivo* studies of freshly harvested human vascular rings *ex vivo*. It is unlikely that this difference was due to *JCAD* mediated changes in systemic inflammation, since no difference in either IL-6, TNF-α, or CRP levels was observed between *JCAD* genotypes. This study is the first to show that the JCAD protective allele is associated with improved endothelial cell function. Improved vasodilation was also observed in *Jcad* knockout mice fed a HFD but not in mice fed a chow diet,^12^ indicating that this interaction is associated with other cardiovascular disease risk factors. The *JCAD* protective allele is associated with reduced *JCAD* expression, as demonstrated by Jones *et al.*^3^ (2018) who showed that the lead CAD-associated SNP (rs2487928) is a highly significant eQTL for *JCAD* expression in aortic and internal mammary artery tissue and Xu *et al*.^12^ who observed the same association for rs9337951. These data indicate a protective effect of loss of *JCAD* on endothelial cell function. Future studies investigating how loss of *JCAD* alters conduit and resistance artery function will be key to understand the mechanisms mediating these findings. All participants in this study had pre-existing cardiovascular disease; it would be interesting to establish the role of JCAD in vascular function in health individuals.

To test the causal role of reduced *JCAD* expression on the progression of atherosclerotic plaque formation, we crossed *Jcad*^−/−^ mice onto the *ApoE*^−/−^ background. Loss of *Jcad* results in a dramatic decrease in plaque development in both the aortic root and on the inner curvature of the aortic arch, both areas associated with a low or disturbed pattern of blood flow.^13^ This work complements the recent study by Xu *et al*.^12^ where loss of *Jcad* was shown to reduce atherosclerosis burden in both the aortic root and aorta and expanded this finding to show the loss of *Jcad* in endothelial cells alone was sufficient to mediate this effect. The finding that loss of *Jcad* is associated with reduced plaque burden by two independent groups using two different *Jcad* knock out mouse lines demonstrates the robustness of these findings. We further found that the specific reduction in plaque in the inner curvature was associated with reduced expression of the pro-atherogenic adhesion molecules Icam and Vcam-1. This observation in *ApoE^+/+^* mice was not due to difference in underlying plaque burden. These results indicate that *Jcad* mediates the expression of inflammatory adhesion molecules in areas of pro-atherogenic flow. Furthermore, in a second model of altered flow, hind limb ischaemia, loss of *Jcad* substantially altered the flow-induced recovery response, indicating a role for *Jcad* in the shear stress mechanotransduction pathway. This was confirmed using an *in vitro* shear stress system where siRNA mediated knock down of *JCAD* resulted in decreased activation of downstream regulators of the shear stress mechanotransduction pathway in endothelial cells.

Circulating lipid levels in particular LDL correlate with atherosclerosis progression.^14^ However, as *Jcad* deletion had no effect on plasma levels of either total, LDL or HDL cholesterol or triglycerides it is unlikely that altered lipid levels were responsible for the reduced plaque burden observed in *Jcad*^−/−^ mice, consistent with the recent study by Xu *et al*.^12^ Similarly, the difference in plaque formation was unlikely to be due to differences in blood pressure, as we found no difference in systolic blood pressure between control and *Jcad*^−/−^ mice on either a hyperlipidaemic *ApoE*^−/−^ or wild type (*ApoE^+/+^*) background. These observations are in keeping with recent blood pressure GWAS studies.^15^

The finding in this study of a significant reduction in the expression of Vcam-1 and Icam-1 in the inner curvature of the aortic arch in *Jcad*^−/−^ mice is consistent with the results from two previous studies were siRNA knock down of *JCAD* in endothelial cells resulted in a significant reduction in VCAM-1 and ICAM-1 expression.^3^^,^^12^ Endothelial cell expression of VCAM-1 and ICAM-1 trigged by disturbed flow is one of the earliest events in atherosclerosis development preceding the presence of other markers of atherosclerosis such as monocyte recruitment.^16^^,^^17^ It is anticipated that the reduction in pro-inflammatory adhesion molecules in *Jcad*^−/−^ mice would result in a significant reduction in monocyte recruitment to the vessel wall. Indeed, loss of *JCAD* leads to a significant decrease in monocyte adhesion in LPS and TNF-α simulated endothelial cells.^3^^,^^12^

The finding that loss of *Jcad* leads to a significant reduction in both Vcam-1 and Icam-1 in the inner curvatures, an area consistently associated with disturbed flow, indicates that *Jcad* may play a role in the response to pathological flow. In a second model of altered vascular flow, namely the hind limb ischaemia model, we show that loss of *Jcad* leads to impaired recovery of blood flow to the ischaemic limb. Recovery from hind limb ischaemia is critically dependent on both arteriogenesis and angiogenesis. Downstream of the occlusion site the resultant ischaemic insult activates hypoxia sensitive pathways and the release of growth factors such as VEGF leading to the proliferation and migration of endothelial cells and tube formation.^18^ In our study, the reduced recovery of limb perfusion at later time points is most likely due to reduced angiogenesis. Loss of *Jcad* is associated with reduced tumour angiogenesis^5^ and impaired VEGF signalling in endothelial cells, with reductions in VEGF mediated proliferation, migration, and tube formation.^3^^,^^5^

Changes in haemodynamic forces after femoral artery ligation, such as fluid shears stress, drives endothelial cell activation, monocyte recruitment, and outward collateral remodelling.^19^ The acute increase in flow through the collateral vessels post-occlusion leads to a shear stress mediated activation of NK-КB leading to up-regulation of VCAM-1 and ICAM-1 in endothelial cells.^20^ Disruption of this pathway results in inadequate outward collateral remodelling and reduced recovery.^21^ As reduced recovery was apparent in *Jcad*^−/−^ mice from as early as 7 days post-ligation and *JCAD* has been found to co-localize with VE-Cadherin in endothelial cells,^4^ which forms part of the junctional mechanosensor complex,^22^^,^^23^ we hypothesized that this reduced recovery was in part due to disruption of shear stress mediated collateral remodelling. We tested this hypothesis using an *in vitro* model of shear stress onset in primary human endothelial cells. Knock down of *Jcad* was associated with a blunted activation of the shear stress mechanotransduction pathway with reduced phosphorylation of AKT, eNOS, and NF-КB p65. This indicates that loss of *Jcad* disrupts the shear stress mechanotransduction pathway. The reduced atherosclerosis burden in *Jcad*^−/−^ mice could also be due to increased activity of the Hippo pathway. YAP/TAZ has been implicated in the shear stress mechanotransduction pathway with laminar shear stress found to inhibit YAP/TAZ activity by modulation of integrin Gα13 RhoA pathway.^24^^,^^25^ JCAD could modulate the Hippo pathway by either interacting with LATS2, a negative regulator of the Hippo pathway,^3^ or via interaction with actin-binding proteins. Xu *et al*.^12^ have suggested that the JCAD driven pro-atherogenic phenotype is drive by direct interactions between JCAD and actin binding partners such as TRIOBP to regulate F-actin dependent YAP/TAZ activation. In this study, we used a global knock out mouse and hence cannot exclude the possibility that loss of *Jcad* in alternative cell types such as vascular smooth muscle cells or monocyte/macrophages could play a causative role.

We observed both beneficial and detrimental vascular effects of loss of JCAD, depending on the vascular pathologies. Both progression of atherosclerosis and recovery from hind limb ischaemia are critically determined by alterations in haemodynamic forces. In the *Jcad* knock out mouse alteration in the mechnotrasduction pathway are atheroprotective. In contrast in the model of hind limb ischaemia, lack of *Jcad* leads to impaired recovery. Targeting *Jcad* for therapeutic benefit will require a detailed understanding of the mechanistic and temporal role of *Jcad* in these and other vascular pathologies.

## 5. Conclusion

We have shown for the first time that *JCAD* is a novel CAD susceptibility gene, mediated by altered responses to changes in endothelial cell shear stress sensing. Identifying this new role for *JCAD* in atherosclerotic plaque progression highlights the importance of new CAD genes that mediate blood flow mechanotransduction in the pathogenesis of CAD, and as potential novel targets for treatments to reduce atherosclerotic plaque formation, independent of established risk factors, and biologic mechanisms.

## Authors’ contributions

Concept: G.D., H.W., T.K., E.T and K.M.C. Carried out experiments and analysis: G.D., V.M., A.A.H.Z., I.A., T.K., V.S.R., S.C., L.T., L.D., E.D., A.G and C.A. Wrote the manuscript: G.D and K.M.C.

**Conflict of interest:** none declared.

## Funding

This work was supported by the British Heart Foundation (Project Grant PG/15/35/31403, Programme Grants RG/17/10/32859, RG/12/5/29576, senior fellowship FS/16/15/32047, and Chair Award CH/16/1/32013), Wellcome Trust (090532/Z/09/Z), BHF Centre of Research Excellence, Oxford (RE/13/1/30181 and RE/18/3/34214), and the National Institute for Health Research (NIHR) Oxford Biomedical Research Centre.

## Supplementary Material

cvz263_Supplementary_Material

## Notes

Translational perspective We reveal that *JCAD* is a novel coronary artery disease susceptibility gene which determines atherosclerosis progression via a role in the endothelial cell shear stress mechanotransduction pathway. Identifying this new role for *JCAD* in atherosclerotic plaque progression highlights the importance of new coronary artery disease genes that mediate blood flow mechanotransduction in the pathogenesis of coronary artery disease. These genes are potential novel targets for treatments to reduce atherosclerotic plaque formation, independent of established risk factors and biological mechanisms.
