# Supplementary material for: A key role for the novel coronary artery disease gene JCAD in atherosclerosis via shear stress mechanotransduction
Source: Cardiovasc Res. 2019 Oct 4;116(11):1863–74. doi: 10.1093/cvr/cvz263 (PMC7449560; doi:10.1093/cvr/cvz263)
Supplement: cvz263_Supplementary_Material [file cvz263_supplementary_material.pdf]

Supplementary Data

A key role for the novel coronary artery disease gene *JCAD* in atherosclerosis via shear stress mechanotransduction

Gillian Douglas[1, 2,*], Vedanta Mehta[1, 2], Ayman Al Haj Zen[1, 2, 3], Ioannis Akoumianakis[1], Anuj Goel[1, 2], Victoria S. Rashbrook[1, 2], Lucy Trelfa[1, 2], Lucy Donovan[1, 2], Edward Drydale[1, 2], Surawee Chuaiphichai[1, 2], Charalambos Antoniades[1], Hugh Watkins[#1, 2] Theodosios Kyriakou[#1, 2], Ellie Tzima[#1, 2] and Keith M. Channon[#1, 2]

[#] = Authors jointly supervised this work

[1]BHF Centre of Research Excellence, Division of Cardiovascular Medicine, Radcliffe Department of Medicine, John Radcliffe Hospital, University of Oxford, UK,

[2]Wellcome Centre for Human Genetics, University of Oxford, Roosevelt Drive, Oxford, UK

[3]College of Health and Life Sciences, Hamad Bin Khalifa University, Education City, Qatar Foundation, Doha, Qatar

Supplementary Table 1: Demographic characteristics of study participants

	**rs2487928 AA**	**rs2487928 AG**	**rs2487928 GG**
Participants (n)	97	200	146
Age (years)	66.7±1.0	66.6±0.8	66.8±0.8
Males (%)	78.3	84.5	78.1
Hypertension (%)	68.0	71.0	69.2
Hyperlipidaemia (%)	72.2	77.5	73.3
T2DM (%)	20.6	22.5	23.9
Smoking			
Active (%)	6.2	12.6	14.4
Past (%)	56.7	52.8	49.3
BMI (kg/m^2)	29.0±0.4	27.9±0.3	28.7±0.3
**Medication**			
Antiplatelet (%)	78.4	84.5	78.1
ACEi/ARBs (%)	72.2	59.5	60.3
Statins (%)	82.5	83.0	80.8
β-blockers (%)	67.0	66.5	61.6
Calcium channel blockers (%)	26.8	25.0	28.8
Insulin (%)	12.4	6.0	7.6
Oral hypoglycaemic (%)	14.4	15.5	17.1

T2DM: Type 2 diabetes mellitus; BMI: Body mass index; ACEi: Angiotensin converting enzyme inhibitor; ARB: Angiotensin receptor blocker; continuous variables are presented as mean ± standard error of the mean (SEM)

Supplementary Table 2: Body and Organ Weights

	Jcad$^{+/+}$ *ApoE*$^{-/-}$	*Jcad*$^{+/-}$ *ApoE*$^{-/-}$	*Jcad*$^{-/-}$ *ApoE*$^{-/-}$
Body weight	25.1±0.7	25.9±0.6	27.2±0.8
Heart	0.17±0.01	0.18±0.01	0.21±0.01
Kidney	0.31±0.02	0.31±0.01	0.31±0.04
Liver	1.72±0.10	1.74±0.12	1.85±0.20
Lung	0.18±0.01	0.17±0.02	0.23±0.04
Spleen	0.14±0.01	0.12±0.01	0.13±0.02
n	15	10	6

Kidney weight is the combined weight of both kidneys. Data presented as the mean±SEM

Supplementary Table 3: Plasma lipid levels

	Jcad$^{+/+}$ *ApoE*$^{-/-}$	*Jcad*$^{+/-}$ *ApoE*$^{-/-}$	*Jcad*$^{-/-}$ *ApoE*$^{-/-}$
Total cholesterol (mmol/L)	33.9±1.1	31.2±2.5	34.2±1.9
LDL cholesterol (mmol/L)	9.9±0.7	9.6±0.9	9.1±0.1
Triglycerides (mmol/L)	2.1±0.1	2.2±0.2	2.0±0.2
HDL cholesterol (mmol/L)	2.6±0.2	2.6±0.2	2.6±0.2
n	22	16	7

Data presented as the mean±SEM

Supplementary Figure 1

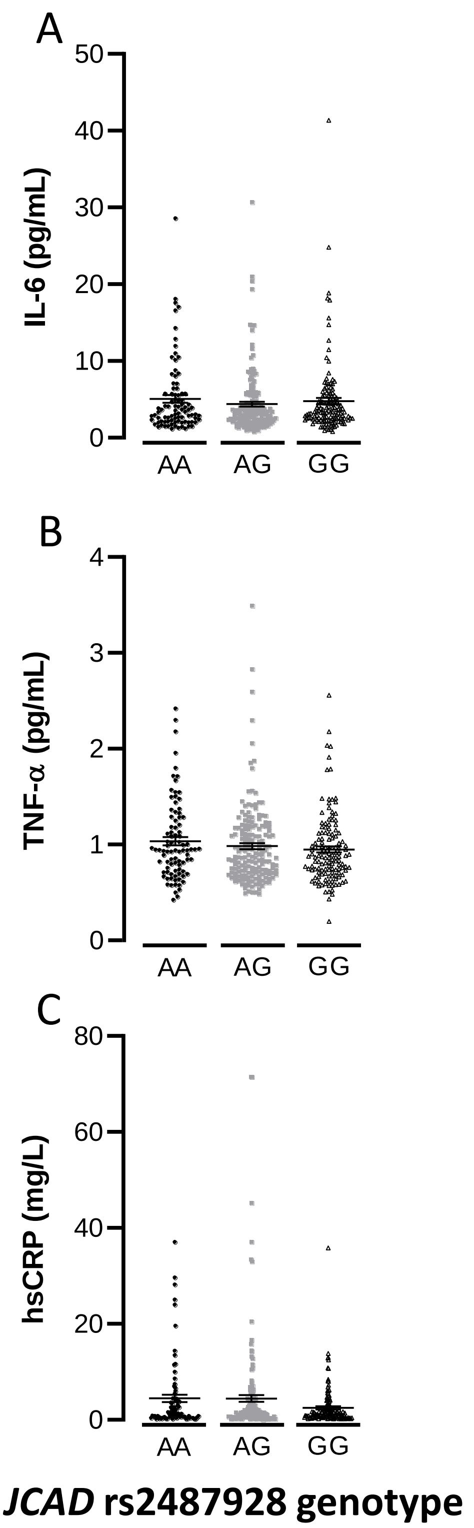

Supplementary Figure 1: Markers of systemic inflammation were not different in carriers of the risk allele (AA) compared with carriers of the protective (GG) allele. A) IL-6 (number of subjects: AA=88, AG=173, GG=133), B) TNF-α (number of subjects: AA=89, AG=176, GG=134) and C) hsCRP (number of subjects: AA=89, AG=177, GG=134) one-way ANOVA for repeated measures.

Supplementary Figure 2

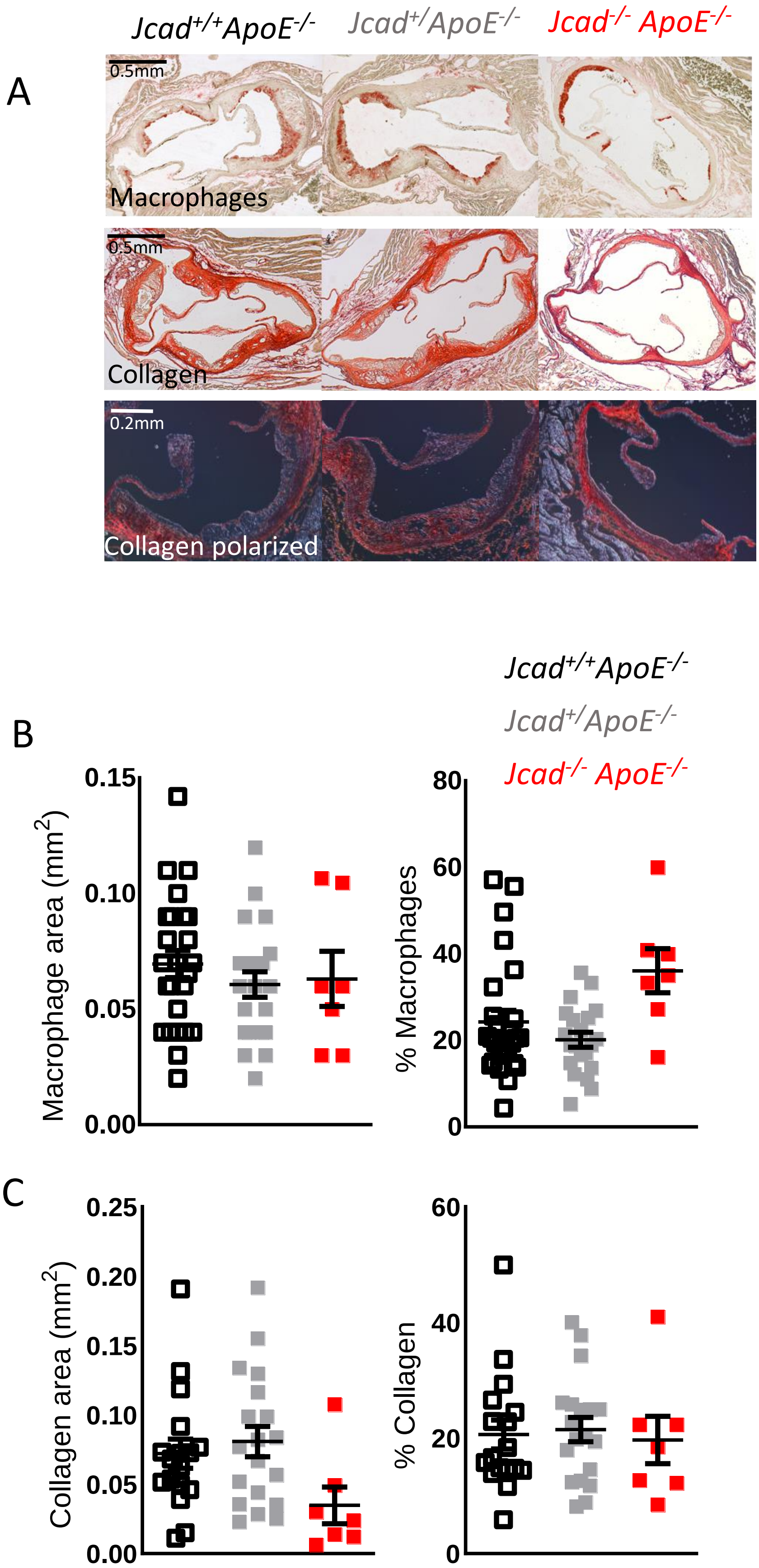

Supplementary Figure 2: Loss of *Jcad* does not alter plaque macrophage collagen content in aortic root plaques from wild type (*Jcad*$^{+/+}$ *ApoE*$^{-/-}$*),* heterozygous (*Jcad*$^{+/-}$ *ApoE*$^{-/-}$) and homozygous (*Jcad*$^{-/-}$ *ApoE*$^{-/-}$) knock out mice fed a high fat diet for 10 weeks. A) Representative images of aortic roots stained for macrophages (Galetin-3, macrophages stain red, scale bar = 0.5mm) and collagen (Sirius red, collagen stains red, scale bar = 0.2mm). Quantification of total (B) macrophage and (C) collagen content in aortic roots. No differences were observed in either macrophage or college content between genotypes P>0.05 one-way ANOVA or Kruskal-Wallis test). Data are expressed as the mean±SEM, each date point represents an individual mouse. Black symbols = *Jcad*$^{+/+}$ ApoE$^{-/-}$, grey symbols = *Jcad*$^{+/-}$ ApoE$^{-/-}$, red symbols = *Jcad*$^{-/-}$ ApoE$^{-/-}$.

Supplementary Figure 3

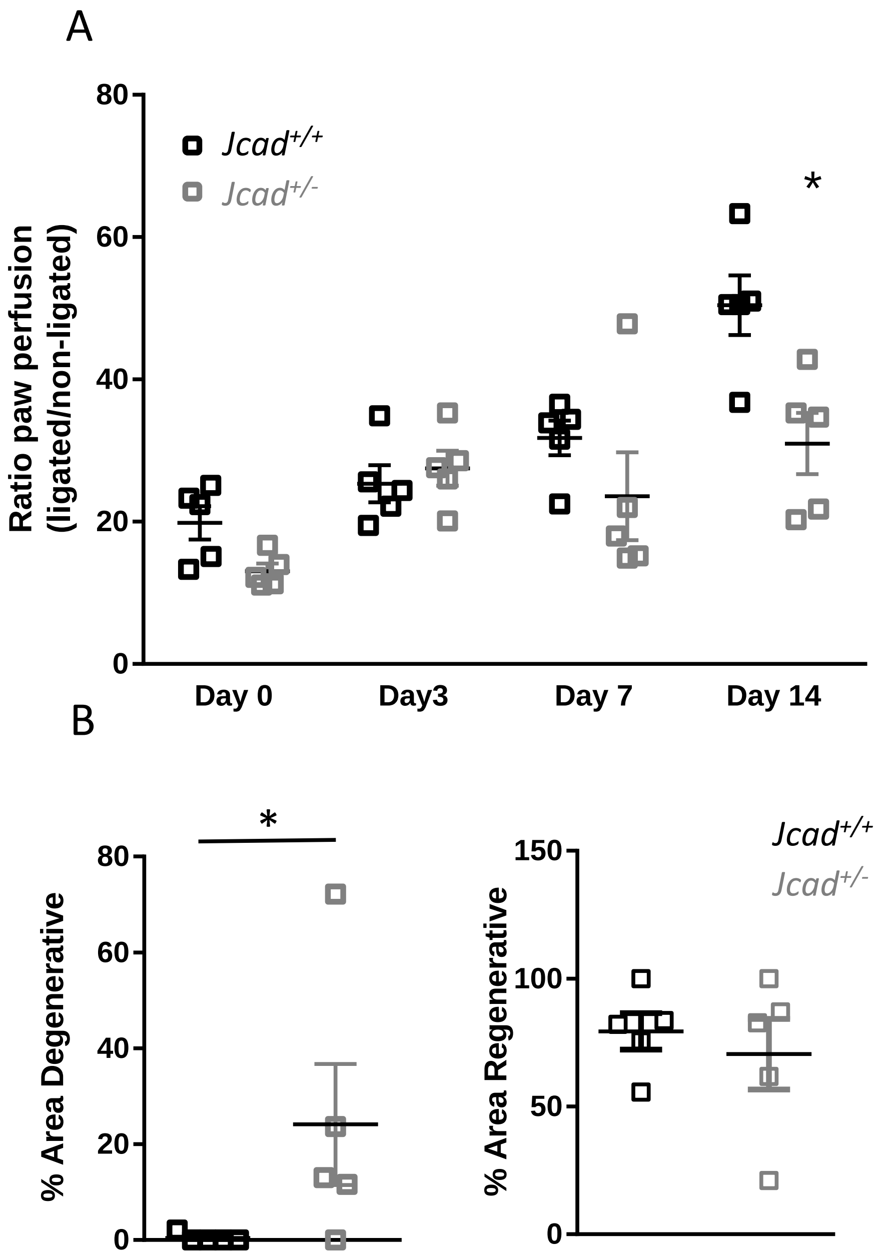

Supplementary Figure 3: *Jcad* Heterozygous knock out mice (*Jcad*$^{+/-}$) have a reduced recovery after hind limb ischemia. A) Reduced perfusion recovery of plantar perfusion in *Jcad*$^{+/-}$ mice after femoral artery ligation compared with wild type (*Jcad*$^{+/+}$) littermates (*=P<0.05, RM ANOVA*).* C) Loss of *Jcad* is associated with an increased presence of degenerative tissue (defined as hyperesoniphillic muscle with no or swollen nuclei and the presence of multiple cellular infiltrate) but no difference in the area of regenerative tissue (defined as the presence of centralised nuclei) in gastrocnemius muscle 14 days after femoral artery ligation (*=P<0.05, Mann-Whitney). Data are expressed as the mean±SEM, each point represent an individual animal. Black symbols = *Jcad*$^{+/+}$, grey symbols = *Jcad*$^{+/-}$.
